# Factors associated with self-care behavior in patients with chronic kidney disease: a systematic review

**DOI:** 10.1186/s12882-025-04137-9

**Published:** 2025-04-25

**Authors:** Mella Riski, Irma Melyani Puspitasari, Cherry Rahayu, Sofa D. Alfian

**Affiliations:** 1 Department of Pharmacology and Clinical Pharmacy, Faculty of Pharmacy, Universitas Padjadjaran, Sumedang, West Java 45363 Indonesia; 2 Center of Excellence for Pharmaceutical Care Innovation, Universitas Padjadjaran, Jatinangor, Indonesia; 3 Dr. Hasan Sadikin Central General Hospital, Bandung City, West Java 40161 Indonesia; 4 Center for Health Technology Assessment, Universitas Padjadjaran, Jatinangor, Indonesia

**Keywords:** Chronic kidney disease, Factors, Self-care behavior

## Abstract

**Background:**

Chronic kidney disease (CKD) is a significant global health issue associated with cardiovascular risk, elevated morbidity and mortality rates, reduced quality of life, and high medical costs. Self-care behavior (SCB) is an effective strategy for mitigating the negative impacts of CKD. Identifying factors that influence SCB in CKD patients is essential for improving clinical outcomes. This study analyzes the factors affecting self-care behavior in patients with CKD.

**Methods:**

A structured search was conducted on PubMed and EBSCO up to June 10th, 2024. This review was not limited by publication year, published in English, and only full-text articles were included.

**Results:**

A total of 510 articles were identified from both databases. After removing 109 duplicates, 401 articles remained. Sixteen articles met the inclusion criteria. The results showed that several factors were associated with SCB, including health literacy (HL), social support, disease knowledge (DK), age, occupation, income, marital status, place of residence, gender, education, comorbidities, smoking habits, body mass index, participation in CKD programs, duration since CKD diagnosis, CKD stage, psychological factors, therapy compliance, self-efficacy, and laboratory results (triglyceride, PCR urine, hemoglobin, phosphor, and albumin levels).

**Conclusions:**

The findings indicated that multiple factors can influence SCB in patients with CKD. The most factors that showed a significant association with SCB were age and education in 5 studies, respectively. These findings underscore the importance of addressing patient-specific factors to improve patient SBC through education and counseling from healthcare providers.

**Supplementary Information:**

The online version contains supplementary material available at 10.1186/s12882-025-04137-9.

## Background

Chronic kidney disease (CKD) is a disorder of kidney structure or function that occurs for > 3 months and has an impact on health [1]. CKD is a global health problem associated with cardiovascular risk, high morbidity and mortality rates, decreased quality of life, and high medical costs [2–4]. In 2017, CKD sufferers globally were recorded at 697.5 million people and caused 1.2 million deaths [5]. According to the World Health Organization (WHO), the global death rate from CKD is estimated to increase by around 14% by 2030 [6].

Early detection and treatment of CKD can delay disease progression [7]. Self-care behavior (SCB) is the ability to manage life with chronic illness, which includes monitoring the condition, treatment adherence, and responding to maintain quality of life. SCB is associated with better clinical outcomes in chronic diseases to reduce the adverse effects associated with CKD [2, 4]. Identifying factors influencing SCB in CKD patients helps improve clinical outcomes [8]. SCB in CKD is associated with improved physical function, decreased urinary protein, and controlled blood pressure [9]. SCB in CKD includes therapy compliance, diet, exercise, smoking habits, and blood pressure monitoring [4]. In addition, SCB may include therapy adherence, physical activity, smoking habits, and BMI control. Long-term therapy adherence is important to avoid disease progression and life-threatening complications [10]. Physical activity is associated with improved cardiovascular outcomes in CKD patients. Whereas, quitting smoking and maintaining a normal body mass index (BMI) has been shown to reduce the incidence of proteinuria, which is one of the signs of kidney damage [2].

Several patient demographics, including age, gender, marital status, education, occupation, income comorbidities, and laboratory data, are correlated with SCB [3, 4, 7]. One of the factors related to SCB is health literacy (HL) [4]. However, the relationship between HL and SCB is not always consistent because SCB may be influenced by other factors, such as knowledge of the disease [2]. Poor disease knowledge (DK) contributes to inadequate SCB, which is a barrier to efficient CKD management [3]. The results of the study showed that there was a positive correlation between SCB and DK in CKD patients [3].

Previous systematic reviews have not focused on discussing factors related to SCB in patients with CKD. Previous studies only discussed the relationship between health literacy and self-management in CKD patients [11]. In addition, other studies discussed self-efficacy training interventions in hemodialysis patients [12] and self-management related to reducing urinary protein, blood pressure levels, exercise capacity, and CRP levels [13]. Other studies used qualitative studies related to barriers and facilitators to kidney disease management [14]. Systematic reviews specifically addressing factors associated with SCB in CKD patients remain limited. Therefore, this review analyzes factors related to SCB in CKD patients, which are expected to be a consideration for health care providers to improve SCB patients with CKD by considering related factors.

## Methods

This study was conducted in accordance with the Preferred Reporting Items for Systematic Reviews and Meta-Analyses (PRISMA) 2020 guideline [15]. The PRISMA checklists of this study are presented in Supplementary Materials (Table S3).

### Search strategy

A structured article search was conducted in PubMed and EBSCO up to June 10th, 2024. The search strategy in PubMed used Medical Subject Headings (MeSH) and “title/abstract” after keywords. In addition, use the connectors “OR” and “AND” to combine several keywords. Search strategy on PubMed, namely ((“Associated Factors“[Title/Abstract] OR “Influencing Factors“[Title/Abstract] OR “Predictors“[Title/Abstract] OR “Correlates“[Title/Abstract] OR “Obstacles“[Title/Abstract] OR “Challenges“[Title/Abstract] OR “Barriers“[Title/Abstract] OR “Indicators“[Title/Abstract] OR “Difficulties“[Title/Abstract] OR “Causes“[Title/Abstract] OR “Factors“[Title/Abstract] OR “Determinants“[Title/Abstract] AND (fft[Filter])) AND (“Self Care“[Mesh] OR “Self-Care“[Mesh] OR “Self Management“[Mesh] OR “Self-Management“[Mesh] OR " Self Care“[Title/Abstract] OR “Self-Care“[Title/Abstract] OR “Self Management“[Title/Abstract] OR “Self-Management“[Title/Abstract] OR “Self Care Behavior“[Title/Abstract] OR “Self-Care Behavior“[Title/Abstract] OR “Self Care Behaviour“[Title/Abstract] OR “Self-Care Behaviour“[Title/Abstract] OR “Self Care Behaviors“[Title/Abstract] OR “Self-Care Behaviors“[Title/Abstract] OR “Self Care Behaviours“[Title/Abstract] OR “Self-Care Behaviours“[Title/Abstract] OR “Self Care Behavior Scale“[Title/Abstract] OR “Self-Care Behavior Scale“[Title/Abstract] OR “Self Care Behaviour Scale“[Title/Abstract] OR “Self-Care Behaviour Scale“[Title/Abstract] AND (fft[Filter]))) AND (“Chronic Kidney Disease“[Title/Abstract] AND (fft[Filter])).

Search strategy on EBSCO, namely S1 AND S2 AND S3. S1: associated factors OR influencing factors OR predictors OR correlates OR obstacles OR challenges OR barriers OR indicators OR difficulties OR causes OR factors OR determinants). S2: (self care or self-care) OR (self management or self-management) OR (self care behavior or self-care behavior) OR (self care behaviour or self-care behaviour) OR (self care behaviors or self-care behaviors) OR (self care behaviours or self-care behaviours) OR (self care behavior scale or self-care behavior scale) OR (self care behaviour scale or self-care behaviour scale). S3: chronic kidney disease. Search strategies in PubMed and EBSCO are available in the Supplementary Materials (Tables S1 and S2).

### Inclusion and exclusion criteria

Inclusion criteria include patients with CKD as the primary diagnosis, primary focus on factors related to self-care behavior (SCB) or self-care (SC) or self-management (SM), full-text articles, published in English, and no restrictions on publication year. Exclusion criteria included qualitative study, bookshelf, commentaries, letters to the editor, and editorials because of the tendency to reflect individual opinions or responses to previous publications, compared to presenting original investigations. Study protocols and abstracts from conference proceedings were excluded due to lacking rigor and comprehensive data necessary for systematic review.

### Study selection

The articles acquired from the search results were entered into Mendeley version 2.122.0 (Elsevier, RELX, NY, USA) to identify any duplicates. One researcher (MR) reviewed the titles and abstracts to identify articles relevant to the keywords. Articles without titles and abstracts pertinent to self-care or self-management in chronic kidney disease (CKD) patients were excluded. Furthermore, all articles that potentially met the inclusion criteria were thoroughly examined, resulting in the identification of studies focused on self-care or self-management issues in CKD patients. This review process was conducted by MR. Articles that met the inclusion criteria were then validated by SDA, IMP, and CR.

### Data extraction and review process

Articles that met the inclusion criteria were extracted into Microsoft Excel 2021 version 2408 (Microsoft Corporation, Redmond, WA, USA). One researcher (MR) independently extracted data related to title, author, year of publication, country, study site, population, study design, survey instrument, respondent characteristics data (gender, CKD stage, and dialysis status), and research results related to factors affecting SC or SM of CKD patients. Other researchers (SDA, IMP, and CR) independently verified the extracted data. The review results are discussed by all researchers to obtain consistent results, and decisions are reached by consensus.

### Risk of bias assessment

This review uses 2 tools to assess risk of bias (RoB). The tool used to assess RoB in non-randomized studies is Newcastle-Ottawa Scale [16]. This tool consists of 3 domains, namely selection, comparability, and outcome. The difference in tools used in cross-sectional studies consists of 7 items, while cohort studies consist of 8 items. Meanwhile, the tool used to assess RoB in Randomized Controlled Trials (RCTs) is the Jadad Scale. This tool consists of 3 domains, including randomization, blinding, and withdrawals and dropouts, with a score range of 0–5. A total score of 5 points determines the quality assessment, with 0–2 points indicating low quality and 3–5 points indicating high quality [17].

### Strengh of evidence

Assessment of the quality of evidence requires the validity of individual study results for important outcomes [18].

## Results

**Fig. 1:**
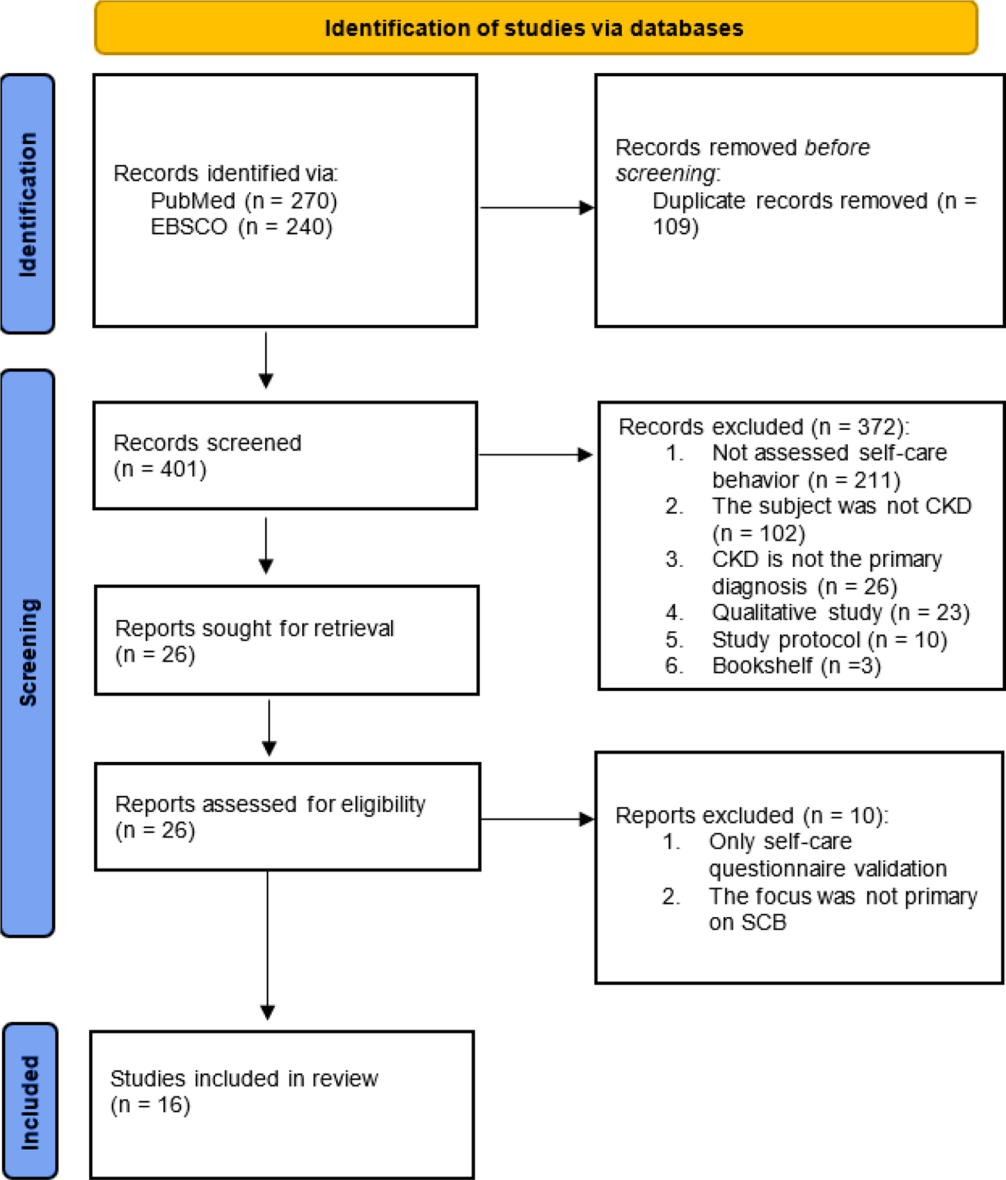
PRISMA flowchart: the study selection

Based on the PubMed and EBSCO database searches (Fig. 1), 270 and 240 articles were obtained. After removing duplicates, 401 articles were obtained. The excluded articles were those that did not assess self-care/self-management (211 articles), subjects were not CKD patients (102 articles), CKD was not the primary diagnosis (26 articles), qualitative studies (23 articles), protocol studies (10 articles), and bookshelf (3 articles). The final results obtained were 16 articles that were reviewed.

Characteristics of the studies are shown in Table 1.

**Table 1:** Characteristics of the studies

Characteristics	Number of Studies
Study Design:	
Cross-sectional Cohort RCT	14 1 1
Years:	
2016 2017 2018 2019 2020 2021 2022 2023	1 0 1 1 0 7 4 2
**Participants**:	
50–100 101–200 201–300 301–400 > 400	1 6 6 0 3
**Stage of CKD**	
1–3 1–4 1–5 2–4 3–5 End Stage Renal Disease	2 1 8 1 1 3
**Dialysis Status**:	
Non-dialysis Non-dialysis and hemodialysis Hemodialysis Peritoneal dialysis Unknown	7 1 4 1 2
**Instrument**:	
CKD Self-Management-29 (CKDSM-29) CKD Self-Care (CKDSC) scale CKDSC-J CKD Self-Management index (CSI) Chronic Disease Self-Management Program (CDSMP) Hemodialysis Self-Management Instrument (HDSMI) Self-Care Behavior Self-Care Scale	5 3 1 1 1 1 1 1
**Factors**:	
Age Education level Health literacy (HL) Comorbidity Social support Occupation Body mass index (BMI) Marital status Laboratory result Duration of CKD diagnosis Gender Disease knowledge (DK) Income Smoking status Health education session Stage of CKD Psychology Treatment adherence Self-efficacy Resilience Illness perception Duration of dialysis Living status	7 7 4 4 4 3 3 3 3 3 2 2 2 2 2 2 2 1 1 1 1 1 1

**Table 2:** Description of research that discusses factors related to SCB in CKD patients

Reference	Year	Country	Participant	Study Design	Instrument	Patient Characteristic				
						Age (year)	Gender (%)		Stage of CKD	Dialysis status
							Female	Male		
Wang et al. [19]	2019	Taiwan	449	Cross-sectional	CKDSC	63.9 ± 13.1	43	57	1–5	Non-dialysis
Yu et al. [4]	2021	Taiwan	208	Cohort	CKDSC	63.2 ± 12.8	41.3	58.7	1–5	Non-dialysis
Wembenyui et al. [24]	2021	Australia	77	Cross-sectional	CKDSM-29	67.2 ± 13.19	49.4	50.6	1–4	Non-dialysis
Suarilah & Lin [8]	2022	Indonesia	226	Cross-sectional	CKDSM-29	56.61 ± 7.48	66.37	33.63	1–3	Non-dialysis
Almutary & Tayyib [22]	2022	Saudi Arabia	203	Cross-sectional	CKDSM-29	47.3 ± 12.1	49.8	50.2	3–5	Non-dialysis
Cardol et al. [25]	2023	Netherland	460	Cross-sectional	CKD self-management index (CSI)	58.5 ± 12.5	37.6	62.4	2–4	Non-dialysis
Kajiwara & Morimoto [26]	2023	Japan	212	Cross-sectional	CKDSC-J	64.9 ± 12.9	34	66	1–5	Non-dialysis
Ahn et al. [20]	2022	South Korea	278	Cross-sectional	CKDSC	57.04	45.3	54.7	1–5	Non-dialysis and dialysis
Lai et al. [23]	2021	Taiwan	112	Cross-sectional	CKDSM-29	70.16 ± 11.59	38.39	61.61	1–3	Dialysis
Washington et al. [27]	2016	USA	107	Cross-sectional	Chronic Disease Self-Management Program (CDSMP)	63 ± 8	49	51	End Stage Renal Disease	Hemodialysis
Noviana & Zahra [28]	2021	Indonesia	107	Cross-sectional	Hemodialysis Self-Management Instrument (HDSMI)	-	40	60	End Stage Renal Disease	Hemodialysis
Kim & Cho [29]	2021	South Korea	100	Cross-sectional	Self-care behavior	51.70 ± 9.40	39	61	End Stage Renal Disease	Hemodialysis
Avanji et al. [30]	2021	Iran	201	Cross-sectional	Self-care Scale	53.4 ± 12.8	56.7	43.3	1–5	Hemodialysis
Chen et al. [32]	2021	China	101	RCT	Self-management scale	48.67 ± 3.44	43.56	56.44	1–5	Peritoneal Dialysis
Chen et al. [21]	2018	Taiwan	410	Cross-sectional	CKDSM-29	70.43 ± 13.10	36.8	63.2	1–5	-
He et al. [31]	2022	China	131	Cross-sectional	Chronic Disease Self-Management Behavior Scale	-	52.67	47.33	1–5	-
			Total = 3382							

The year of publication of the 16 articles was in the period 2016–2023 (Table 2). From 16 articles, a total of 3382 CKD patients were obtained. The age range of participants from 16 articles was 47–70 years. Of the 16 articles, CKD patients who participated in the study comprised 45.18% females and 54.82% males.

**Table 3:** Risk of bias for non-randomized study (Newcastle-Ottawa scale)

References	Risk of Bias (RoB)
Wang et al. [19]	LQ
Yu et al. [4]	HQ
Wembenyui et al. [24]	HQ
Suarilah & Lin [8]	HQ
Almutary & Tayyib [22]	HQ
Cardol et al. [25]	HQ
Kajiwara & Morimoto [26]	LQ
Ahn et al. [20]	HQ
Lai et al. [23]	LQ
Washington et al. [27]	LQ
Noviana & Zahra [28]	LQ
Kim & Cho [29]	LQ
Avanji et al. [30]	LQ
Chen et al. [21]	LQ
He et al. [31]	LQ

LQ = Low quality, HQ = High quality

In this study, 15 studies were assessed using Newcastle-Ottawa Scale consisting of 14 cross-sectional studies [8, 19–30, 31] and one cohort study [4]. The results of the assessment using the Newcastle-Ottawa Scale (Table 3) obtained 6 studies with high quality and 9 studies with low quality.

**Table 4:** Risk of Bias for randomized controlled trials (Jadad scale)

References	Randomization	Blinding	Withdrawals and dropouts	Total Score
Chen et al. [32]	2	0	1	3/5

One study was assessed using the Jadad Scale (Table 4) [32]. The total score obtained was 3, which indicates that this study is of high quality.

**Table 5:** Grading for strength of evidence [18]

Strength of Evidence	Criteria
Strong	≥ 2 high-quality studies showing positive association between the presence of a variable and the outcome AND No studies showing a negative association
Moderate	One high-quality AND one lesser-quality study showing association AND No studies showing negative association
Weak	> 2 low-quality studies showing positive association OR Only one high-quality study showing positive association
Negative	≥ 1 high-quality study showing negative association (inverse relationship) AND No studies showing a positive association
Inconclusive	Associations present in only low-quality study OR No studies of any quality showing univariate association OR Presence of positive and negative associations from different articles, regardless of study quality

**Table 6:** Strength of evidence

Variables	Results						Strength of Evidence
	Significant				No Significant		
	Positive association		Negative association				
	LQ	HQ	LQ	HQ	LQ	HQ	
Age	[12, 20, 25]	[4]		[15]	[23]	[13]	Inconclusive
Education level	[12, 16, 25]	[4, 8]			[22]	[15]	Strong
Health literacy (HL)	[14]	[4, 8, 13]					Strong
Comorbidity			[23]	[4, 8]	[12]		Negative
Social support	[14, 21, 22]	[24]					Strong
Occupation	[25]	[13]		[4]			Inconclusive
Body mass index (BMI)			[12]	[4, 15]			Negative
Marital status	[12]	[17]				[4]	Moderate
Laboratory result				[4, 24]		[13]	Negative
Duration of CKD diagnosis		[4]		[15]	[12]		Inconclusive
Gender	[12]			[4]			Inconclusive
Disease Knowledge				[15]		[17]	Negative
Income		[8]			[25]		Weak
Smoking status		[13]		[4]			Inconclusive
Health education session	[12]	[4]					Moderate
Stage of CKD	[12]	[13]					Moderate
Psychology	[16]	[18]					Moderate
Treatment adherence	[22]						Inconclusive
*Self-efficacy*		[17]					Weak
Resilience	[23]						Inconclusive
Illness Perception	[19]						Inconclusive
Duration of dialysis					[22]		Inconclusive
Living status						[13]	Inconclusive

LQ = Low quality, HQ = High quality

The results of the assessment of the strength of evidence (Table 6) obtained 3 strong studies, 4 moderate studies, 2 weak studies, 4 negative studies, and 10 inconclusive studies.

**Table 7:** Factors associated with SCB in CKD patients

Factors	References	Significant Results	No Significant Results
Age (*n* = 7)	Ahn et al., [20]		Age ≥ 65 years was not significantly correlated with SCB (β = 0.14, *p* = 0.059).
	Wang, et al., [19]	The self-care score was 4.83 points (*p* < 0.001) higher for patients older than 65 years than for younger patients.	
	Almutary & Tayyib, [22]	Age was negatively correlated with SCB (β = −0.183; *p* = 0.004).	
	Washington et al., [27]	Patients older than 70 were significantly associated with the self-management behavior of fluid adherence (*p* = 0.04).	
	Avanji et al., [30]		Age was not significantly correlated with SCB (β = − 0.11, *p* = 0.005)
	He et al., [31]	Age was significantly associated with the disease cognition domain (F = 3.664; *p* = 0.028).	Age was not significantly associated with the domain of treatment management (F = 1.797, *p* = 0.170), exercise behaviors (F = 0.989, *p* = 0.375), diet behaviors (F = 1.000, *p* = 0.371), emotional management (F = 1.099, *p* = 0.336), and self-management knowledge (F = 1.868, *p* = 0.159).
	Yu et al., [4]	● Age was positively correlated with exercise domain (β = 0.05, *p* = 0.03). ● Age was negatively correlated with the home blood pressure monitoring domain (β = -0.04, *p* = 0.02).	Age was not significantly correlated with the domain of diet (β = -0.01, *p* = 0.59), smoking habits (β = 0.03, *p* = 0.07), medication adherence (β = 0.03, *p* = 0.34) and total SCB (β = 0.05, *p* = 0.4).
Education level (*n* = 7)	Wang, et al., [19]	The self-care score was 3.238 points (*p* = 0.033) higher for college and above than for no degree.	
	Almutary & Tayyib, [22]		High school or less was not significantly correlated with SCB (β = 0.023; *p* = 0.705).
	Lai et al., [23]	Having an education level of senior high school or above increased the odds ratio for having high self-management to 4.47 (p-values < 0.05).	
	Suarilah & Lin [8]	The level of education showed a significant association with self-management (t = 3.25, *p* = 0.023).	
	Kim & Cho, [29]		College or above (t = 1.80, *p* = 0.076) and high school (t = 1.69, *p* = 0.095) was not significantly associated with SCB
	He et al., [31]	Education was significantly associated with the emotional management domain (F = 7.165; *p* = 0.004).	Education was not significantly associated with the domain of treatment management (F = 1.236, *p* = 0.309), exercise behaviors (F = 2.025, *p* = 0.155), diet behaviors (F = 1.016, *p* = 0.378), disease cognition (F = 1.236, *p* = 0.309) and self-management knowledge (F = 0.207, *p* = 0.815).
	Yu et al., [4]	Senior high school or above was correlated with the domain of diet (β = 1.82, *p* = 0.001), home blood pressure monitoring (β = 1.70, *p* < 0.001), and medication adherence (β = -1.42, *p* = 0.04).	Senior high school or above was not correlated with the domain of exercise (β = 0.99, *p* = 0.08), smoking habits (β = -0.43, *p* = 0.27) and total SCB (β = 2.65, *p* = 0.08).
Health literacy (HL) (*n* = 4)	Yu et al., [4]	● HL was positively correlated with the domain of diet (βadj = 0.15, *p* < 0.005), exercise (βadj = 0.11, *p* = 0.004) and total SCB (βadj = 0.27; *p* = 0.008). ● The patients with sufficient or excellent health literacy had higher diet (β = 1.80, *p* = 0.003), exercise (βadj = 1.22, *p* = 0.02), and home blood pressure monitoring (βadj = 1.03, *p* = 0.03) performance than those with inadequate or limited/problematic health literacy.	
	Chen et al., [21]	HL was positively correlated with SCB (*r* = 0.33; *p* < 0.001).	
	Suarilah & Lin [8]	● HL showed a positively significant correlation with total self-management (*r* = 0.536), self-integration (*r* = 0.457), problem-solving (*r* = 0.528), and seeking social support (*r* = 0.381). ● HL (*r* = − 0.200) negatively correlated with adherence to the recommended regimen.	
	Ahn et al., [20]	HL (Actively managing my health) was correlated with SCB (β = 0.41; *p* < 0.001).	
Comorbidity (*n* = 4)	Suarilah & Lin [8]	Family history of comorbidity (F = 6.28, *p* = 0.013) showed a significant association with SM.	
	Avanji et al., [30]	Being diabetic was associated with SCB (β=-0.09; *p* = 0.01).	
	Yu et al., [4]	Patients with diabetes mellitus were correlated with the home blood pressure monitoring domain (β = -0.80, *p* = 0.08).	● Patient with hypertension was not significantly correlated with the domain of diet (β = -0.43, *p* = 0.41), exercise (β = -0.51, *p* = 0.34), home blood pressure monitoring (β = 0.07, *p* = 0.88), smoking habits (β = 0.24, *p* = 0.52), medication adherence (β = -0.49, *p* = 0.47) and total SCB (β = -1.13, *p* = 0.44). ● Patient with diabetes mellitus was not significantly correlated with the domain of diet (β = 0.27, *p* = 0.62), exercise (β = -0.63, *p* = 0.25), smoking habits (β = 0.25, *p* = 0.53), medication adherence (β = 0.13, *p* = 0.85) and total SCB (β = -0.79, *p* = 0.60). ● Patient with heart disease was not significantly correlated with the domain of diet (β = 0.50, *p* = 0.46), exercise (β = -0.69, *p* = 0.31), home blood pressure monitoring (β = -0.20, *p* = 0.73), smoking habits (β = -0.31, *p* = 0.52), medication adherence (β = 0.23, *p* = 0.79) and total SCB (β = -0.47, *p* = 0.80).
	Wang et al., [19]		The number of comorbidities was not a significant difference in self-care score among 2–3 comorbidities (*p* = 0.412), and 4 + comorbidities than 0–1 comorbidities (*p* = 0.705).
Social support (*n* = 4)	Kim & Cho, [29]	Social support increased SCB (β = 0.56, *p* < 0.001).	
	Chen et al., [21]	● Social support was correlated with SCB (*r* = 0.64; *p* < 0.001) ● Social support from healthcare providers (*r* = 0.60) and family (*r* = 0.50) was strongly correlated with SCB	
	Noviana & Zahra, [28]	There was a significant relationship between high social support and self-management (*p* = 0.027, odds ratio = 2.386).	
	Chen et al., [32]	After the 6-month intervention, the self-management ability of the intervention group was significantly different from that of the control group (*P* < 0.01).	
Occupation (*n* = 3)	He et al., [31]	The occupation was associated with the domain of treatment management (F = 1.770; *p* = 0.032), exercise behaviors (F = 12.019; *p* = 0.001), diet behaviors (F = 6.032; *p* = 0.015), and disease cognition (F = 7.370; *p* = 0.008).	Employed status was not significantly associated with the domain of emotional management (F = 0.201, *p* = 0.655) and self-management knowledge (F = 1.897, *p* = 0.171).
	Ahn et al., [20]	Not working was correlated with SCB (β = 0.16, *p* = 0.037).	
	Yu et al., [4]	The worker was correlated with the domain of exercise (β = -1.24, *p* = 0.02), smoking habits (β = -0.98, *p* = 0.008) and total SCB (β = -2.60, *p* = 0.07).	The worker was not significantly correlated with the domain of diet (β = -0.07, *p* = 0.89), home blood pressure monitoring (β = 0.44, *p* = 0.33), and medication adherence (β = -0.75, *p* = 0.28).
Body mass index (BMI) (*n* = 3)	Wang, et al., [19]	The self-care score was 2.24 points lower for patients with a BMI ≥ 24 kg/m^2^ than BMI ≤ 24 kg/m^2^ (*p* = 0.005).	
	Almutary & Tayyib, [22]	BMI was negatively associated with SCB (β = −0.159; *p* = 0.010).	
	Yu et al., [4]	BMI was correlated with the domain of diet (β = -0.15, *p* = 0.02), home blood pressure monitoring (β = -0.12, *p* = 0.04) and total SCB (β = −0.38, *p* = 0.04).	BMI was not significantly correlated with the domain of exercise (β = -0.12, *p* = 0.07), smoking habits (β = -0.02, *p* = 0.71), and medication adherence (β = 0.03, *p* = 0.74).
Marital status (*n* = 3)	Wang, et al., [19]	The self-care score was 1.826 points (*p* = 0.0411) higher for married or partnership than for single.	
	Yu et al., [4]		Married was not significantly correlated with the domain of diet (β = -0.42, *p* = 0.51), exercise (β = 0.36, *p* = 0.58), home blood pressure monitoring (β = -0.40, *p* = 0.46), smoking habits (β = -0.05, *p* = 0.92), medication adherence (β = -0.72, *p* = 0.39) and SCB (β = -1.22, *p* = 0.49).
	Wembenyui et al., [24]	Levels of CKD self-management were significantly higher in patients who were married than those who were not (*p* < 0.01).	
Laboratory result (*n* = 3)	Yu et al., [4]	Log-transformed triglycerides (β = −7.91, *p* = 0.01) and log-transformed urine PCR (β = −2.45, *p* = 0.04) were significantly negatively correlated with SCB.	The laboratory result was not significantly correlated with SCB: • eGFR (ml/min/1.73m2) (β = 0.01, *p* = 0.71) • Hemoglobin (g/dl) (β = 0.13, *p* = 0.72) • Albumin (g/dl) (β = 1.74, *p* = 0.29) • Uric acid (mg/dl) (β = 0.93, *p* = 0.02) • Cholesterol (mg/dl) (β = -0.00, *p* = 0.93) • Log-formed glycated hemoglobin (β =-1.17, *p* = 0.06)
	Chen et al., [32]	● There were significant differences between the WeChat group and the face-to-face group for hemoglobin and blood phosphorus (*p* < 0.05). ● There were significant differences in albumin, hemoglobin, blood phosphorus, and calcium levels between the intervention and control groups after 3 months and 6 months of intervention (*p* < 0.01).	
	Ahn et al., [20]		The laboratory result was not significantly correlated with SCB: • Serum hemoglobin ≥ 11 g/dl (within range) (β = -0.06, *p* = 0.426) • Serum calcium < 8.5 or > 10.2 mg/dl (β = -0.03, *p* = 0.69) • Serum creatinine > 1.3 mg/dl (β = 0.06, *p* = 0.717) • Serum eGFRd ≥ 60 ml/min/1.73m^2^ (within range) (β = 0.16, *p* = 0.382).
Duration of CKD diagnosis (*n* = 3)	Yu et al., [4]	CKD duration was positively correlated with total SCB (β = 0.51, *p* = 0.004) and exercise domain (β = 0.26, *p* < 0.001)	CKD duration was not significantly correlated with the domain of diet (β = 0.05, *p* = 0.49), home blood pressure monitoring (β = 0.03, *p* = 0.54), smoking habits (β = 0.07, *p* = 0.12), and medication adherence (β = 0.10, *p* = 0.25).
	Almutary & Tayyib, [22]	Time aware of CKD 0–12 months (less than a year) was associated with SCB (β = −0.050; *p* = 0.405).	
	Wang et al., [19]		Duration of CKD diagnosis was not significantly different in self-care score among 3–4 years (0.424 points, *p* = 0.742) and 5 + years (1.327 points, *p* = 0.234) than < 3 years.
Gender (*n* = 2)	Yu et al., [4]	The female was correlated with the domain of exercise (β = -1.36, *p* = 0.01), medication adherence (β = -1.35, *p* = 0.04) and total SCB (β = −3.27, *p* = 0.02).	The female was not significantly correlated with the domain of diet (β = -0.60, *p* = 0.25), home blood pressure monitoring (β = -0.27, *p* = 0.54), and smoking habits (β = -0.32, *p* = 0.39).
	Wang et al., [19]	Self-care score was 2.00 points (*p* = 0.020) higher for females than for males.	
Disease Knowledge (*n* = 2)	Almutary & Tayyib, [22]	DK was negatively correlated with SCB (β = − 0.513; *p* < 0.0001).	
	Wembenyui et al., [24]		CKD knowledge was not significantly correlated with CKD self-management behavior (*r* = 0.02, *p* = 0.90).
Income (*n* = 2)	Suarilah & Lin [8]	Monthly income (F = 6.14, *p* = 0.014) was a significant association with the total score for self-management.	
	He et al., [31]		Income was not significantly associated with the domain of treatment management (F = 1.975, *p* = 0.143), exercise behaviors (F = 0.461, *p* = 0.632), diet behaviors (F = 1.99, *p* = 1.141), emotional management (F = 0.127, *p* = 0.881), disease cognition (F = 2.130, *p* = 0.446), and self-management knowledge (F = 0.812, *p* = 0.446).
Smoking status (*n* = 2)	Yu et al., [4]	Smokers were negatively correlated with SCB (β = −9.10, *p* = 0.001).	Smoker was not significantly correlated with the domain of diet (β = -1.69, *p* = 0.08), exercise (β = -1.20, *p* = 0.23), home blood pressure monitoring (β = -1.05, *p* = 0.20), and medication adherence (β = -0.89, *p* = 0.48).
	Ahn et al., [20]	Not smoking was correlated with SCB (β = 0.32; *p* = 0.002).	
Health education session (*n* = 2)	Wang, et al., [19]	Participating in the CKD integrated care program had significantly higher self-care scores than no participation (*p* < 0.001).	
	Yu et al., [4]	The number of health education sessions was correlated with the domain of exercise (β = 0.10, *p* < 0.001), smoking habits (β = 0.03, *p* = 0.04) and total SCB (β = 0.21, *p* = 0.001).	The number of health education sessions was not significantly correlated with the domain of diet (β = 0.02, *p* = 0.39), home blood pressure monitoring (β = 0.01, *p* = 0.75), and medication adherence (β = 0.06, *p* = 0.05).
Stage of CKD (*n* = 2)	Wang, et al., [19]	CKD stage 2 or higher had significantly higher self-care scores (*p* < 0.001).	
	Ahn et al., [20]	ESRD was correlated with SCB (β = 0.3; *p* = 0.004).	
Psychology (*n* = 2)	Cardol et al., [25]	● CSI total was significantly correlated with psychological distress (β_adj_ = 1.04, *p* = 0.001), depressive symptoms (β_adj_ = 1.09, p = < 0.001), and anxiety symptoms (β_adj_ = 1.06, *p* = 0.018). • Psychological distress was negatively associated with domain dietary adherence (β_adj_ = − 0.13, *p* = 0.006), physical activity (β_adj_ = − 0.13, *p* = 0.01), and medication adherence (β_adj_ = − 0.15, *p* = 0.002). ● Depressive symptoms were negatively associated with domain dietary adherence (β_adj_ = − 0.14, *p* = 0.004), physical activity (β_adj_ = − 0.15, *p* = 0.002), and medication adherence (β_adj_ = − 0.15, *p* = 0.002). ● Anxiety symptoms were negatively associated with domain dietary adherence (β_adj_ = − 0.11, *p* = 0.03) and medication adherence (β_adj_ = − 0.13, *p* = 0.009).	• Psychological distress was not significantly associated with domain body mass index (β_adj_ = 0.09, *p* = 0.07) and smoking (β_adj_ = 1.04, *p* = 0.13). • Depressive symptoms were not significantly associated with domain body mass index (β_adj_ = 0.08, *p* = 0.09) and smoking (β_adj_ = 1.07, *p* = 0.07). • Anxiety symptoms were not significantly associated with domain body mass index (β_adj_ = 0.05, *p* = 0.35), smoking (β_adj_ = 1.04, *p* = 0.38), and physical activity (β_adj_ = − 0.07, *p* = 0.19).
	Lai et al., [23]	Depression was associated with SM (0.246; *p* = 0.03).	
Treatment adherence (*n* = 1)	Kim & Cho, [29]	Treatment adherence (t = 5.94, *p* < 0.001) was significantly associated with SCB.	
*Self-efficacy* (*n* = 1)	Wembenyui et al., [24]	A positive relationship was found between CKD self-management and self-efficacy (*r* = 0.37, *p* < 0.01), with high levels of self-efficacy associated with high levels of CKD self-management.	
Resilience (*n* = 1)	Avanji et al., [30]	Resilience was associated with SCB (β = 0.78, *p* = 0.001).	
Illness Perception (*n* = 1)	Kajiwara & Morimoto, [26]	Illness perception was associated with SCB (F = 7.31, *p* < 0.001).	
Duration of dialysis (*n* = 1)	Kim & Cho, [29]		Duration of dialysis was not significantly associated with SCB (t = − 0.66, *p* = 0.514)
Living status (*n* = 1)	Ahn et al., [20]		Living status with family or someone was not significantly associated with SCB (β = 0.04, *p* = 0.644).

Factors associated with SCB in patients with CKD are shown in Table 7. Based on the results of a review of 16 articles (Table 7), factors that were significantly associated with domain or total SCB include age (*n* = 5), education level (*n* = 5), HL (*n* = 4), comorbidity (*n* = 3), social support (*n* = 4), occupation (*n* = 3), body mass index (BMI) (*n* = 3), marital status (*n* = 2), laboratory result (*n* = 2), duration of CKD diagnosis (*n* = 2), gender (*n* = 2), DK (*n* = 1), income (*n* = 1), smoking status (*n* = 2), health education session (*n* = 2), stage of CKD (*n* = 2), psychology (*n* = 2), treatment adherence (*n* = 1), *self-efficacy (n* = 1), resilience (*n* = 1), and illness perception (*n* = 1). The most factors that showed a significant association with SCB were age and education in 5 studies, respectively.

## Discussion

This study has revealed that among 16 studies on SCB in patients with CKD, factors significantly associated with the domain or total SCB included, age, education level, health literation (HL), comorbidity, social support, occupation, body mass index (BMI), marital status, laboratory result, duration of CKD diagnosis, gender, disease knowledge, income, smoking status, health education session, stage of CKD, psychology, treatment adherence, *self-efficacy*, resilience, and illness perception.

Five studies showed a significant association between age and SCB [4, 19, 22, 27, 31]. Self-care scores were higher in patients ≥ 65 years of age compared to younger patients [19]. This result is similar to the study by Tsai et al. (2021), which showed a relationship between age and SCB. Older age has better SCB. This may be because awareness of poor physical function and various morbidities can encourage older patients to have better SCB [3]. However, research by Almutary & Tayyib (2022) shows that age is negatively correlated with SCB [22]. This may be because with increasing age, physical condition decreases and they have lower SCB energy than younger patients [33–36]. Two studies showed no significant correlation between age and SCB [20, 30].

Education is one of the factors that influences SCB in CKD patients. Five studies showed a significant relationship between education and SCB [4, 8, 19, 23, 31]. Education level significantly affects self-management, self-integration, and problem-solving [8]. Education up to college shows a significant influence on overall self-management. This finding is in line with research by Van Prooijen [37] and Schunk & DiBenedetto [38]; education level affects various cognitive, emotional, and social outcomes, and having much better problem-solving skills. Nevertheless, two studies obtained no significant results between education and SCB [22, 29]. The higher the level of education and income, the higher the tendency of individuals to join and actively participate in social networks such as the chronic kidney disease community [28]. Education is a strong predictor of HL because patients with higher levels of education are more likely to be able to understand, interpret, and evaluate information than patients with lower levels of education [4].

HL was significantly correlated with SCB [4, 8, 20, 21]. A higher total HL score correlates significantly with better SCB [4]. Higher HL levels can increase patient confidence in their ability to manage their disease, thus positively affecting SCB [4]. The study by Zhong et al. [39] demonstrated similar findings, specifically a positive correlation between health literacy and self-care behavior. Nevertheless, studies by Wong et al. (2018) and Schrauben et al. [40] did not find a positive correlation between HL and SCB in CKD patients. The inconsistency of some studies may be due to only focusing on the relationship between functional HL (such as reading and writing) and SCB [41, 42]. Health-related reading and writing abilities are not always associated with understanding in decision-making [43].

Three studies showed a significant relationship between comorbidity and SCB [4, 8, 30]. Patients with diabetes were significantly associated with SCB [30]. Comorbidity is a potential factor that influences self-integration and adherence to recommended regimens [8]. Kim & Eaton’s (2017) research showed similar results, where patients without comorbidities demonstrated better self-integration [44]. These findings are similar to Seo’s research [45], which indicated that comorbidities such as hypertension, diabetes, anemia, and obesity, along with worsening renal function, can exacerbate pre-existing comorbidities. However, one study showed that there was no significant difference in the number of comorbidities that patients had [19].

Four studies showed that social support was significantly associated with SCB [21, 28, 29, 32].

The higher the level of patient social support, the higher the fulfilment of treatment, which can be interpreted as a contribution to improving SCB [29]. Patients who were given a social support intervention for 6 months obtained significant results associated with self-management skills [32]. Support in managing the disease is important to encourage managing the disease and making health decisions [46]. Healthcare providers are an important source of social support for patients to learn SCB [21, 28]. Healthcare providers can provide support through ongoing assessment of patient readiness to manage their illness [24]. Family members play a role in helping patients manage their illness and are expected to assist in encouraging SCB, thereby ensuring better health outcomes [21, 28]. Wittenberg et al. [47] showed that SCB in CKD patients is a complex condition and is greatly influenced by the social support to achieve disease management goals.

Three studies showed an association between occupation and SCB [4, 20, 31]. Patients who did not work were positively correlated with SCB [20]. These findings are similar to the study conducted by Tsai et al. (2021), which demonstrated a correlation between work and SCB. Employed patients exhibit lower SCB than their unemployed counterparts. Patients engaged in employment may encounter greater challenges in executing SCB owing to work schedules or occupational stress [3, 9].

BMI was significantly correlated with SCB [4, 19, 22]. The self-care score was lower for patients with a BMI greater than 24 kg/m^2^ [19]. BMI was negatively correlated with SCB [22]. This may be because patients are more confident and aware and adhere to treatment plans that include diet and daily physical activity, which are factors in controlling patient weight [22]. Schrauben et al. [40] indicate that obesity may elevate the chance of developing chronic kidney disease (CKD).

The self-care score was higher for married couples or partnerships than for singles [19]. CKD self-management was significantly higher in married patients than for unmarried patients [24]. The study by Gheewala et al. (2018) demonstrated that married patients had superior CKD knowledge ratings in comparison to single or unmarried individuals. This may be attributed to those who lived with others being more active in acquiring health-related information and adopting a healthy lifestyle. Family helps patients manage their illness, thereby ensuring better health outcomes [21, 28]. Nevertheless, one study found no significant association between marital status and total SCB score [4].

Several laboratory results related to patient SCB include triglyceride levels, urine PCR, hemoglobin, phosphorus, and albumin. Two studies showed a significant relationship between laboratory results and SCB [4, 32]. Yu et al.‘s (2021) study showed that serum triglyceride and urine PCR values ​​were negatively correlated with SCB. Patients with higher SCB scores had lower serum triglycerides and UPCR. These results are similar to the study by Tsai et al. (2021), which found that some laboratory results, such as eGFR, were correlated with SCB and DK. However, one study obtained no significant results on hemoglobin, calcium, creatinine, and eGFR [20].

CKD duration was significantly positively correlated with total SCB [4]. Almutary & Tayyib (2022) obtained an association between time awareness of CKD 0–12 months and SCB. Wang et al. (2019) showed no significant difference in self-scare scores between patients undergoing diagnosis for 3–4 years and more than 5 years with patients undergoing dialysis < 3 years. CKD duration was influenced by the number of patient visits to healthcare providers [22]. Wang et al.‘s (2019) research obtained results showing a relationship between gender and SCB. Self-care scores were higher in women than in men [19]. This result differs from research by Yu et al. (2021), which showed that SCB was negatively correlated with female gender.

DK was associated with SCB in non-dialysis patients [22]. Similar results were found in the study by Tsai et al. (2021), which demonstrated a relationship between DK and SCB. Low DK can be influenced by the number of patient visits to healthcare providers and the lack of education related to CKD [22]. Higher levels of knowledge are associated with better SCB [22]. Adequate DK and the ability to apply problem-solving strategies and seek information by reading scientific papers or discussing with health professionals [23]. Patients with high DK tend to be more confident managing their disease [22]. Nevertheless, the results of a study by Wembenyui et al. (2021), showed no relationship between DK and SCB. The results are similar to Schrauben et al.‘s [40] research, which found no relationship between DK and SCB. However, one way to increase self-confidence to be able to manage oneself and comply with the treatment regimen is to increase knowledge about the disease and its treatment [24].

One of the factors that influences SCB is income [8]. Monthly income with a regional minimum wage or more significantly influenced overall self-management and problem-solving abilities [8]. A comparable study of patients with CKD revealed that those with less education and insufficient monthly income struggled to address daily challenges, both in comprehension and the systematic approach [48].

Smoking status was significantly correlated with total SCB scores [4]. Ahn et al. (2022) found a significant association between non-smoking patients and SCB. Research conducted by Schrauben et al. [40] has shown that smoking may lead to adverse clinical outcomes in patients with CKD. Patients with less awareness of the health concerns associated with smoking consider themselves less susceptible to its adverse effects and exhibit lower intent to modify their smoking habits [2].

Participating in the CKD integrated care program had significantly higher self-care scores [19]. Two studies showed a significant association between health education sessions and SCB [4, 19]. This result is similar to the study by Tsai et al. (2021), which found that increasing the duration of CKD education is positively correlated with the SCB score, which can ultimately reduce the risk of rapid decline in kidney function. The CKD management program plays an important role in instructing SCB and providing advice on patients’ health conditions and lifestyles, thereby improving SCB [21]. SCB was positively correlated with the number of health education sessions [4].

ESRD patients undergoing hemodialysis had higher SCB scores [19]. These results are consistent with the study by Wang et al. (2019), which showed that overall SCB scores were higher in late-stage CKD patients. Low SCB scores in early-stage CKD patients may be due to patients not yet showing physical symptoms due to mild kidney damage, so they do not understand their disease [20]. Patients are not aware of the importance of a healthy lifestyle in managing diet and exercise [20]. Patients with ESRD experience a deficiency of erythropoietin production and decreased red blood cell production, resulting in anemia and physical symptoms due to uremia gradually [19]. Therefore, ESRD patients tend to have a higher awareness of the importance of managing CKD routinely and better SCB compared to patients with early-stage CKD [20]. Patients with ESRD often have contact with their regular physicians [27]. Self-management of ESRD requires adherence to a broader and stricter treatment regimen. However, evidence on self-management for patients with early-stage kidney disease is inadequate. To improve active self-management behavior and facilitate decision-making for non-dialysis CKD patients at various stages, specific knowledge related to kidney disease should be assessed and improved [46].

Cardol et al.‘s (2023) research found that psychological distress was significantly related to SCB. Higher psychological distress was significantly associated with poorer dietary compliance, less physical activity, and lower medication compliance. Similar results were for depressive symptoms, while anxiety was only associated with poorer dietary and medication compliance [25]. This finding is similar to the research conducted by Lai et al. (2021), which indicated that depression reduces the probability of elevated SCB [23]. Typical depressive symptoms are pessimistic perceptions and a lack of assessment of one’s ability to engage in SCB [25]. Psychological distress can include negative cognitions, problems with motivation, energy, self-efficacy, concentration, or social withdrawal, all of which can hinder a patient’s ability to engage in good SCB. These results suggest that psychological distress is a potential barrier to self-management [25].

The results of Avanji et al.‘s (2021) study showed that SCB had a significant relationship with medication adherence. Worse conditions for treatment may lead to increased blood pressure in dialysis patients with lower HL [49]. High treatment adherence is significantly associated with a reduced risk of rapid decline in kidney function in CKD patients [2].

Self-efficacy (SE) was positively correlated with SCB scores [24]. Early-stage CKD in Indonesia showed positive SE [8]. Self-efficacy can help patients perform SCB [23]. These results are similar to the study by Schrauben et al. [40], which showed a relationship between self-efficacy and SCB. High self-efficacy is associated with better SCB in CKD patients. An effective way to optimize patient self-efficacy is through gradual experience and understanding. The study by Lee et al. [50] obtained consistent results that self-efficacy is related to health literacy and SCB.

Avanji et al.‘s (2021) study showed that SCB was positively correlated with resilience and negatively correlated with age. Resilience may be an important factor in improving physical and mental health. Ma et al. [51] found a positive correlation between resilience and health-promoting behaviors in HD patients. Resilience results in a sense of purpose in life, better self-esteem, and effective interpersonal relationships. Resilient individuals are able to face difficulties positively. Therefore, resilience should be considered as one of the important factors influencing SCB [30].

SCB was significantly associated with illness perception [26]. These results are in line with the study by Schrauben et al. [40], which showed that perceived knowledge about CKD was consistently associated with SCB even after adjusting for DK and HL. Having a positive perception of the disease is important for patients living with chronic diseases such as CKD. The study by Demir et al. [52] found that health perception was low in patients with low HL. Patients need insight on how to perceive the decision-making process [53]. Kim and Cho (2021) found that the duration of dialysis was not significantly associated with SCB.

The findings of this review indicate that SCB is affected by multiple factors. Factors significantly associated with both SCB domains and total SCB include age, education level, HL, comorbidities, social support, occupation, BMI, marital status, laboratory results, duration of CKD diagnosis, gender, disease knowledge, income, smoking status, health education sessions, CKD stage, psychology, medication adherence, self-efficacy, resilience, and disease perception. Factors that need to be considered are age and education because each has five studies showing significant results related to SCB. CKD management requires patient involvement in SCB to slow the progression of CKD [9, 54]. Participation in SCB is one way to reduce the negative impacts associated with CKD [2]. SCB interventions such as CKD education programs can provide positive outcomes, including symptom improvement, disease knowledge, favorable self-care behaviors, improved quality of life, and controlled blood pressure. Health care providers can identify the needs of CKD patients by providing health education and counseling programs related to SCB according to patient characteristics, especially age and education, to help patients manage their disease [19]. Understanding of CKD can increase patient confidence in SCB [46, 54].

### Strengths of this study

This study has several strengths. First, this study focuses on factors associated with SCB in patients with CKD. These results are important for healthcare providers in assessing patient characteristics and improving SCB. Second, the review of articles is not limited by the year of publication, so the results obtained are not limited by time. Third, the evaluation was conducted on CKD patients in several countries. This can add information related to factors that influence SCB because it allows for different responses from various countries.

### Limitations

The limitation of this study is that it only included articles with full text available and articles published in English. As a result, some important results from omitted studies may not have been evaluated, potentially limiting the comprehensiveness of the overall analysis. In addition, the heterogeneity of the studies, with variability in the study design and SCB assessment instrument, presents challenges in generalizing the findings. Moreover, focusing exclusively on published studies in English may introduce publication bias, as studies with negative or inconclusive results are less likely to be published. Despite the limitations, this review provides information related to factors associated with SCB in CKD patients so that it can be used as a consideration for healthcare providers in assessing and identifying patients in improving patient SCB. Future research may include quantitative and qualitative studies and use other databases to obtain a more comprehensive approach regarding factors influencing SCB in CKD patients.

## Conclusion

The findings indicated that multiple factors can influence SCB in patients with CKD. The most factors that showed a significant association with SCB were age and education in 5 studies, respectively. Healthcare providers can address SCB by offering health education programs or regular counseling to CKD patients, taking into account characteristics that may influence SCB, particularly the patient’s age and educational attainment.

## Electronic supplementary material

Below is the link to the electronic supplementary material.

Supplementary Material 1

## Data Availability

Availability of data and materials: The data presented in this study are available in the manuscript and Supplementary Materials.
